# THE GERONTOLOGIST

**DOI:** 10.1093/geroni/igae098.0907

**Published:** 2024-12-31

**Authors:** Joseph Gaugler

**Affiliations:** University of Minnesota, Minneapolis, Minnesota, United States

## Abstract

N/A

